# Combination of Polymeric Microneedles with Specific Peptide for TNF‑α Sensing Via Noninvasive Detection in Interstitial Fluid

**DOI:** 10.1021/acsomega.6c07406

**Published:** 2026-09-16

**Authors:** Anna Palma, Concetta Di Natale, Daniele Tammaro, Elena Lagreca, Alessia Cugudda, Sara La Manna, Vincenzo Ferraro, Veronica Vespini, Simonetta Grilli, Pier Luca Maffettone, Sara Coppola

**Affiliations:** † Department of Chemical, Materials, and Industrial Production Engineering (DICMaPI), University of Naples Federico II, 80125 Naples, Italy; ‡ Institute of Applied Sciences and Intelligent System (CNR-ISASI), Via Campi Flegrei 34, 80078 Pozzuoli, Naples, Italy; § Department of Pharmacy, University of Naples Federico II, 80131 Naples, Italy; ∥ Department of Engineering and Architecture, 9370 University of Parma, 43124 Parma, Italy

## Abstract

Microneedle (MN)-based biosensors offer a minimally invasive and blood-free approach for real-time monitoring of clinically relevant biomarkers in interstitial fluid (ISF). Pro-inflammatory cytokines such as Tumor Necrosis Factor-α (TNF-α) can reach higher concentrations in ISF than in serum, which makes this fluid particularly sensitive for detecting early local inflammatory responses. Here, we introduce an antibody-free MN biosensor employing a synthetic high-affinity peptide (P52) for TNF-α recognition in ISF. The novelty of the platform lies in the stable immobilization of the P52 peptide directly on mechanically robust polymeric MNs for a scalable three-dimensional fluorescence assay. The results reported show how the limitations commonly associated with translating monoclonal antibody-based molecular recognition could be overcome using a functionalized polymeric microneedle platform. Poly­(lactic-*co*-glycolic acid) MNs were fabricated via micro-molding and subsequently functionalized with the P52 peptide. A simple surface-conjugation protocol was employed, which assured peptide stability, low production costs, and reliable conjugation. Comprehensive morphological, mechanical, and chemical characterizations were performed through SEM and fluorescence microscopy. Quantitative detection was achieved via a sandwich-type fluorescence assay using an FITC-labeled P52 variant, enabling evaluation of capture efficiency in simulated ISF across graded TNF-α concentrations. The platform exhibited a linear response with a limit of detection of ∼0.2 pM (∼3 pg/mL). Peptide immobilization on MNs enhanced binding affinity, shifting the equilibrium dissociation constant from the nanomolar to the picomolar range. The P11 peptide was used as a negative control for sequence-dependent TNF-α recognition. Mechanical testing confirmed the robustness of the functionalized MNs without structural failure. Overall, this peptide-functionalized MN platform represents a robust, sensitive, and scalable solution for point-of-care TNF-α monitoring directly in ISF, paving the way for next-generation minimally invasive and low-cost diagnostic systems that will revolutionize the remote biomedicine solution.

## Graphical abstract

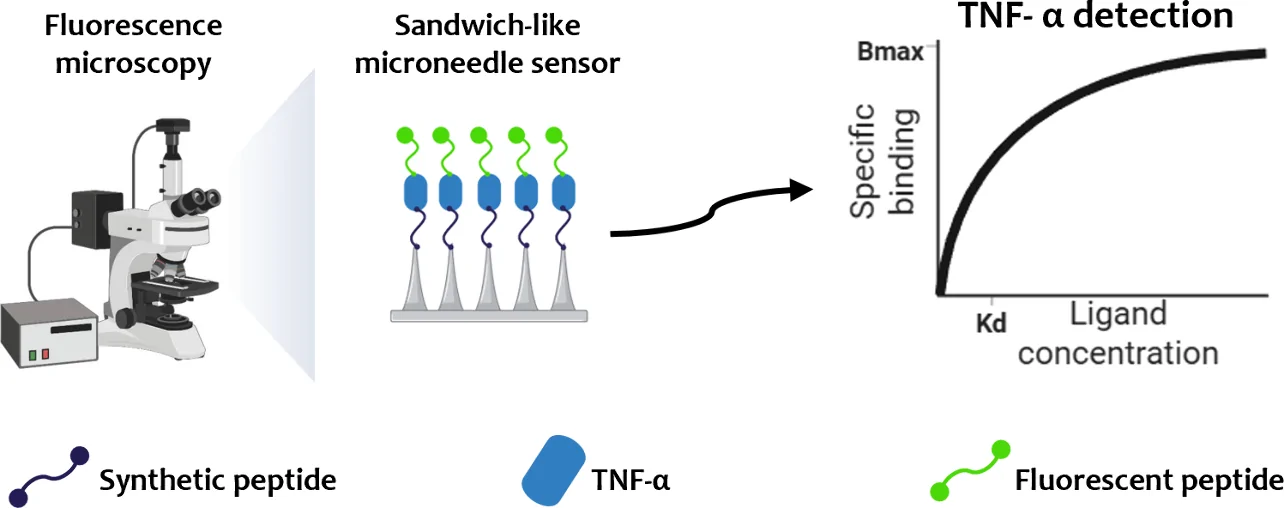

## Introduction

Point-of-Care (PoC) technologies offer the potential for affordable, precise, and real-time diagnostic information. Among these, MN-based biosensors have garnered significant interest as a promising innovation in blood-free diagnostics, due to their minimally invasive and painless access to biomarker-rich ISF. In this context, ISF has garnered significant attention as a promising alternative to blood for minimally invasive sampling. ISF occupies the extracellular space between blood capillaries and tissue cells, playing a central role in the transport of nutrients, hormones, and metabolic products.^1^ Compositionally, it closely resembles blood plasma, containing physiologically relevant analytes such as glucose, lactate, electrolytes, and various molecular biomarkers.^2,3^ These properties, together with ISF low viscosity and its Newtonian fluid behavior under normal physiological conditions, enhance the accuracy of diffusion-based measurements and simplify the modeling of fluid dynamics within the sensing system.^4–7^ In contrast, non-Newtonian biofluids such as blood exhibit shear-thinning and viscoelastic behavior, requiring more complex fluid handling strategies and potentially introducing variability in sensor response.^8–10^ Compared with other peripheral fluids such as sweat, saliva, urine, and tears, ISF shows a superior correlation with blood biomarkers due to its role as an intermediary fluid directly involved in tissue exchange in various pathological conditions, including neuroinflammation associated with tumor progression.^1,2,11,12^ This is further supported by evidence showing that pro-inflammatory cytokines like Tumor Necrosis Factor (TNF) are present at significantly higher concentrations in human ISF than in serum, highlighting ISF’s enhanced sensitivity for detecting local inflammatory responses soon after acute events.^1^ Moreover, ISF is readily accessible just beneath the stratum corneum, enabling potentially localized, repeated, or continuous sampling through minimally invasive platforms such as MNs, with minimal discomfort and without compromising skin integrity.^13^ Polymeric MNs, especially those fabricated from biodegradable polymers such as PLGA, are extensively employed for minimally invasive access to ISF.^14–17^ PLGA MNs combine excellent biocompatibility, tunable mechanical properties, and controlled hydrolytic degradation, making them suitable for both diagnostic and therapeutic applications.^18–20^ Several approaches have been developed for MN fabrication, including lithography, electro-drawing, 3D printing, and pyro-electric formation of three-dimensional microstructures.^21–25^ Among these methods, micro-molding remains particularly attractive because of its cost-effectiveness, reproducibility, and scalability.^26,27^ By integrating biorecognition elements directly onto the MN surface, the micro-molded structures can be transformed into active biosensing platforms capable of combining transdermal access to ISF with in-situ biomarker detection. Conventional MN-based sensors employ antibodies, enzymes, or aptamers as biorecognition elements due to their high specificity and affinity.^28–34^ However, these biomolecules often suffer from limited stability, high production costs, and batch-to-batch variability.^35–39^ Recently, synthetic peptides have emerged as attractive alternatives, offering improved chemical and thermal stability, lower production costs, and easier functionalization, making them suitable for integration into robust and scalable MN sensing platforms.^40,41^ TNFα is a well-characterized pro-inflammatory cytokine and a key biomarker implicated in various pathological conditions, including acute and chronic inflammatory disorders and neuroinflammatory processes associated with tumor progression.^42,43^ Current TNFα detection technologies include digital nanoparticle-based immunosensors,^44^ surface plasmon resonance (SPR)-based biosensors,^45,46^ and electrochemical aptamer-based platforms,^47^ as summarized in Table 1. Although these technologies can provide high analytical sensitivity, their practical implementation for minimally invasive ISF analysis remains challenging. Electrochemical sensors, although sensitive, often suffer from biofouling due to complex biological matrices,^48^ and limited long-term stability,^49^ which restrict their continuous use *in vivo*. Similarly, SPR-based biosensors, while highly specific and label-free, require bulky and expensive instrumentation, are sensitive to environmental variations such as temperature and refractive index changes, and are not easily adaptable for portable or minimally invasive monitoring systems.^50,51^ Moreover, many existing TNF-α sensing approaches have been validated in serum^52^ and saliva,^53^ whereas the use of short synthetic peptides directly immobilized onto polymeric MN surfaces for TNF-α detection remains poorly investigated. Therefore, a research gap remains in the development of peptide-functionalized MN platforms that combine stable bioreceptor immobilization with sensitive cytokine detection in ISF-like matrices. Accordingly, the aim of this study was to investigate the feasibility of using for the first time the P52-functionalized PLGA MNs as an antibody-free platform for sensitive TNF-α detection in simulated subcutaneous ISF. Specifically, the study aimed to (i) propose a stable EDC/NHS-mediated protocol for P52 immobilization on PLGA MNs; (ii) characterize the morphology and mechanical behavior of the functionalized structures; (iii) evaluate TNF-α detection in PBS and simulated ISF; and (iv) assess the analytical performance, apparent binding behavior, and sequence-dependent recognition of the system. The proposed platform exploits the ease of synthesis, scalability, biochemical stability, and stable reliable conjugation of P52 as potential advantages over conventional monoclonal antibody-based recognition systems.^41,54,55^ The main novelty lies in the stable immobilization of a short synthetic peptide onto a mechanically robust MN architecture. A characterization of microneedle morphology was performed using Scanning Electron Microscopy (SEM) and fluorescence microscopy. Fluorescence microscopy was also used as the detection method, since a dye-labeled P52 variant was employed as the detection probe. TNF-α detection in simulated ISF produced a concentration-dependent fluorescence response, with a Limit of Detection (LOD) of approximately 0.2 pM [∼3 pg/mL], comparable to those summarized in Table 1. Immobilization of P52 on MN surface resulted in an enhanced apparent binding affinity, with the equilibrium dissociation constant (K_D_) shifting from the nanomolar range measured through direct BioLayer Interferometry to the picomolar range observed using the MN-based sandwich assay. The P11 peptide was used as a negative control and supported the sequence-dependent recognition of TNF-α by P52. Moreover, compression and collagen-insertion experiments demonstrated that peptide functionalization did not compromise MN mechanical integrity or penetration capability. Despite the increasing use of peptides in biosensing, their direct integration as recognition elements on polymeric MNs remains comparatively underexplored, particularly for cytokine detection in ISF. Overall, this work bridges peptide-based molecular recognition and microneedle-enabled ISF sensing within a single scalable polymeric platform, providing a basis for the future development of minimally invasive and accessible TNF-α monitoring systems.

**Table 1:** Representative Sensing Platforms Reported for TNF-α Detection

Technology/Study	Reported LOD	Matrix	Transduction	Advantages	Disadvantages	Reference
Nanoparticle-supported rapid electronic detection (NasRED)	0.022 pM (in PBS)	PBS. Serum, Synovial fluid	Digital optoelectronic/plasmonic nanoparticle readout	Rapid and accessible readout; potential point-of-care implementation; digital quantification	Antibody-dependent assay; nanoparticle preparation and reader required; not designed for minimal invasiveness	^44^
Receptor-assisted antibody SPR biosensor	Formal LOD not reported	Buffer	Optical (Plasmonic resonance)	Controlled antibody orientation; label-free	Limited sensitivity at low-picomolar concentrations; no validation in complex biological matrices; limited portability	^46^
Aptamer-based sensor with redox label	∼100 pM	Synthetic urine, saliva	Electrochemical (Voltammetry)	Aptamer specificity; suitable for biofluids	Needs optimization for ISF; limited long-term stability	^47^
Multiplex chemiluminescence assay	0.000373 pM	Serum and plasma	Chemiluminescent ELISA	Subfemtomolar sensitivity; simultaneous cytokine detection	Laboratory plate-based assay; enzyme labels and optical imaging required; not portable	^56^
Liquid crystal–based immunosensor	0.062 pM	Diluted saliva	Polarized optical microscopy	Label-free optical response; simple visual readout; good recovery in saliva	Antibody-based; requires preincubation and polarized optical microscopy	^57^
Aptamer-functionalized SPR/Goos–Hänchen shift sensor	0.001 pM	PBS buffer	Surface plasmon resonance with Goos–Hänchen lateral-shift readout	Label-free; aptamer-based recognition; ultralow experimental LOD; enhanced optical sensitivity	Complex optical setup; demonstrated in controlled buffer; no validation in ISF or complex biological samples	^58^

## Materials and Methods

### Reagents and Materials

Poly­(lactic-*co*-glycolic acid) (PLGA RESOMER RG 504H, 38000–54000 Da, lactide : glycolide = 50 : 50), dimethyl carbonate (DMC, 99% v/v), N-(3-(dimethylamino)­propyl)-N′-ethylcarbodiimide hydrochloride (EDC) / *N*-hydroxysuccinimide (NHS), calcium carbonate buffer and Phosphate Buffered Saline (PBS, pH 7.4), Dulbecco’s Modified Eagle Medium (DMEM) were all purchased from Merck/Sigma-Aldrich, Italy. Ecoflex 00-50 (used for micro-molds) was supplied by Smooth-On, Inc., Macungie, Pennsylvania, USA. Recombinant human TNF alpha protein (Active) (ab259410) was supplied by Abcam, Cambridge, UK. Simulated Subcutaneous Interstitial Fluid was provided by BioChemazone, Alberta, Canada. MES Buffer 0.1 M solution (pH 5.5) was provided by ThermoFisher Scientific. Non-peptonized rat-tail Collagen Type I (5 mg/mL) was provided by ibidi, Gräfelfing, Germany.

### Peptide Synthesis and Labeling (P11, FITC-P11, P52, FITC-P52)

P52 (NH_2_–HAIYPRH–CONH_2_) and P11 (NH_2_–ITPAHMD-CONH_2_) peptides were synthesized by solid-phase peptide synthesis (SPPS) using the 9-fluorenylmethoxycarbonyl/*tert*-butyl (Fmoc/tBu) strategy on Rink-amide resin (substitution 0.57 mmol/g, 25 μmol scale), following established procedures.^59^ For fluorescence experiments, an N-terminal lysine protected with the 4-methyltrityl (Mtt) group was introduced. Selective Mtt deprotection was achieved by repeated short treatments with 1% TFA in DCM containing 5% TIS (2 min each, until the resin turned colorless). After this, fluorescein isothiocyanate (FITC) was conjugated to the ε-amine of the lysine. The resulting sequences were: P52, NH_2_–K­(FITC)-HAIYPRH–CONH_2_, and P11, NH_2_–K­(FITC)-ITPAHMD-CONH_2_.

For BioLayer Interferometry (BLI) experiments, P52 was N-terminally biotinylated using NHS-dPEG12-biotin via NHS-ester chemistry targeting the N-terminal primary amine.

Peptides were purified by reverse-phase high-performance liquid chromatography (RP-HPLC) on a semipreparative C18 column (2.2 × 5 cm), applying a linear gradient of 0.1% trifluoroacetic acid (TFA) in CH_3_CN/0.1% TFA in water (5–70% over 20 min) at a flow rate of 15 mL/min. Peptide purity and identity were assessed by liquid chromatography–mass spectrometry (LC-MS), using a LTQ XL Ion Trap mass spectrometer equipped with an electrospray ionization (ESI) source, coupled to an Ultimate 3000 HPLC system. Purified peptides were lyophilized and stored at −20 °C until use.

### BioLayer Interferometry

For BLI interaction assays, the Sartorius Octet N1 System and streptavidin (SA)-coated biosensor tips (Sartorius Italy S.r.l., Grassina-Bagno a Ripoli (FI), Italy) were used. SA biosensor tips were first hydrated in 1X PBS buffer; then, to achieve ligand immobilization, the tips were dipped, for 300 sec with 1000 rpm agitation, into 4 μL of biotinylated–P52 solution (50 μg/mL). Following peptide loading, each tip was placed in contact, for 300 s with a 1000 rpm agitation speed, with 4 μL of TNFα protein at several concentrations (0.5, 1, 2, and 4 nM). All mathematical manipulations were handled by the Octet Analysis Studio 12.2 software from Sartorius. A 1:1 Langmuir binding model was used for data fitting, and the K_D_ was obtained through the global analysis mode. The experiment was carried out in triplicate to ensure reproducibility.

### Microneedles Fabrication

The MN matrix was fabricated through the micro-molding process. First, master molds were replicated using Ecoflex silicone rubber to produce negative molds. Then, 200 μL of PLGA solution (DMC, 25% w/V) was pipetted into the cavities of Ecoflex molds directly. After 24 h of solvent evaporation at ambient conditions, the matrices were detached and stored for future use. A ZEISS Axio Zoom.V16 stereomicroscope, a Hitachi TM3000 Scanning Electron Microscope (SEM), and a ZEISS Axio Imager.M1m (in both bright and fluorescent fields) were employed for morphological characterization of the matrix. For morphological analysis, 20 MNs were measured from SEM images to determine base diameter, tip diameter, height, and center-to-center spacing. Data are reported as mean ± standard deviation.

### Binding Assay for TNF-α Detection

To prepare the biosensor, MNs were chemically modified following a sandwich-like immunosensor procedure employing the P52 peptide as the recognition element. MNs were first submerged in EDC/NHS solution (0.1 and 0.2 M, respectively, Milli-Q water) for 1 h under gentle stirring (80 rpm) to promote the chemical activation of PLGA carboxylic groups. Then, the activated MN chip was washed three times with Milli-Q water for 5 min, and P52 was immobilized onto the EDC/NHS-activated MN surface by submerging the matrices in a 1 mg/mL solution in PBS 1X (37° C overnight, 100 rpm). Subsequently, matrices were washed three times with PBS for 5 min, and the solutions containing the TNF-α analyte at different concentrations (0, 0.11, 0.23, 0.46, 0.69, 1.84, and 5.74 pM) were used to incubate the matrices for 1 h at room temperature under stirring (80 rpm). We performed two sets of experiments: in the first, the TNF-α was diluted in PBS 1X; in the second, the same concentrations were produced using artificial subcutaneous interstitial fluid (ISF). After three washings in PBS for 5 min, samples were submerged in 1 mL of FITC-P52 solution at 1 mg/mL in PBS 1X (37 °C, 100 rpm, 1 h). Before fluorescence observation, matrices were washed with PBS or ISF three times for 5 min and left to completely dry under a fume hood. As a negative control for the detection assay, the same protocol was implemented by using P11 peptide and incubating TNF-α at 0, 0.1, 1.8, and 5.7 pM in subcutaneous ISF. To evaluate the stability of the EDC/NHS mediated immobilization of the P52 peptide onto the MNs, an activation protocol was performed on the matrices by EDC/NHS reaction, followed by overnight incubation with the fluorescent form of the peptide (FITC-P52) at 37 °C and 100 rpm, at a concentration of 1 mg/mL in PBS. After three 5 min washing steps in PBS, fluorescence images were acquired for the activated matrices, the non-activated matrices incubated with FITC-P52, and the ‘bare’ matrices, i.e., untreated ones. For UV-vis characterization of fluorescent peptide solutions, a Thermo Scientific Multiskan SkyHigh Microplate Spectrophotometer was used.

### Fluorescence Microscopy Imaging of Microneedles

Microneedle arrays were imaged using a ZEISS Axio Imager.M1 upright fluorescence microscope (Carl Zeiss Microscopy, Germany) equipped with an AxioCam MRc5 digital camera (camera identifier: AxioCamMR5_7918). Fluorescence images were acquired using an EC Epiplan-Neofluar 10×/0.25 HD DIC M27 objective, with a numerical aperture of 0.25, and the FITC fluorescence filter set. Images were acquired using an exposure time of 3.4 s, a camera gain setting of 0.5, and 1 × 1 binning. Single-plane images were recorded at a resolution of 2584 × 1936 pixels. The spatial calibration was 0.34 μm/pixel along both the x- and *y*-axes, corresponding to a field of view of approximately 878.6 × 658.2 μm. Pixels were square, with a pixel aspect ratio of 1. All samples included in the same experimental comparison were acquired using identical microscope, objective, filter, camera, exposure, gain, binning, and image-resolution settings. Image visualization and dimensional measurements were performed using ImageJ while maintaining the original spatial calibration. When required for figure preparation, brightness and contrast were adjusted uniformly across the entire image and using the same settings for all directly compared experimental groups. These adjustments were applied exclusively for visualization and did not modify the original images used for quantitative analysis.

### Data Analysis

The fluorescence intensity of the MNs was evaluated using ImageJ, an open-source image processing program developed at the National Institutes of Health (NIH), and the average values over three replicates of the experiment – for which 20 needles were analyzed – were plotted for each TNF-α concentration, generating a calibration curve. The same procedure was performed for both test samples in PBS buffer and test samples in ISF. The experimental data points were fitted using the ‘One site – Specific binding’ model of equation: Y= $Y_{NS}+$ $\frac{B_{\max}\cdot x}{K_{D}+x}$ , where *Y* is the fluorescence intensity, *Y*_*NS*_ the non-specific background signal, *X* the analyte concentration, *B*_*max*_ the maximum specific binding, and K_D_ the equilibrium dissociation constant. Additionally, the linear range was analyzed to determine the sensor’s limit of detection (LOD) and limit of quantification (LOQ). Within this linear range, LOD was calculated as 3.3 × σ / s and LOQ as 10 × σ / s, where σ is the standard deviation of the y-intercept and s is the slope of the regression line.

### Mechanical Strength Characterization

A Zwick Roell dynamometer (model Z010) was used for measuring the mechanical properties of the fabricated matrices. We analyzed the unmodified PLGA MNs (referred to as bare PLGA) and the functionalized ones with P52 (referred to as PLGA + P52). A load cell of 50 kg was employed, and a cylindrical probe was used to apply a compression force along the axial direction of the MNs. The test speed was set to 0.5 mm/s, with a Trigger Load of 0.02 N. The force data recorded by the software as a function of time was plotted against the displacement of the probe. Each group was tested in triplicate (n = 3) to ensure the reliability and reproducibility of the results.

### Penetration in Collagen Matrix

To evaluate MN penetration, a 3D collagen gel was used as a dermal-mimicking matrix following a previously developed method.^60^ Non-peptonized rat-tail Collagen Type I (5 mg/mL) was diluted to 4 mg/mL in PBS. The solution was homogenized by pipetting, pH-adjusted with 1 M NaOH and HCl, and quickly dispensed into fluorodishes to prevent premature gelation. Gels were incubated at 37 °C for 30 min to allow fibrillogenesis, and fiber formation was confirmed by phase-contrast microscopy. MN patches were then inserted under sterile conditions, and Z-stack images were acquired by confocal laser scanning microscopy (Leica TCS SP8 Confocal Microscope, 10×/0.95 NA water-immersion objective, 523× 523 pixels). Collagen fibers were visualized via second harmonic generation (SHG). This platform provides a reproducible system to assess MN penetration dynamics and spatial distribution in a dermal-like environment.

## Results and Discussion

The success of the molding fabrication of the PLGA MN chips was confirmed through both optical and SEM inspection. Figure 1-A1, A2 shows two typical optical microscope images of an MN chip. Figure 1-A1 shows a side view of the MNs over the whole surface of the chip, while that in Figure 1-A2 is a magnified view at a more transversal angle to show qualitatively how the shape replicates quite well between the various microneedles on a single row. Figure 1-B shows a typical SEM image of the same MN chip, revealing a base diameter of (300±15) μm, a tip diameter of (30±5) μm, a height of (380±30) μm, and a center-to-center spacing of (600±40) μm, with values reported as mean ± standard deviation from measurements performed on 20 MNs. The relatively narrow variability observed in the MN dimensions indicates that the micro-molding procedure provided reproducible replication of the master geometry across the array. The combination of a narrow tip and a wider base is particularly relevant for MN performance, since tip sharpness favors tissue penetration, whereas the larger basal cross-section contributes to resistance against axial compression and buckling.^61^

**Figure 1:**
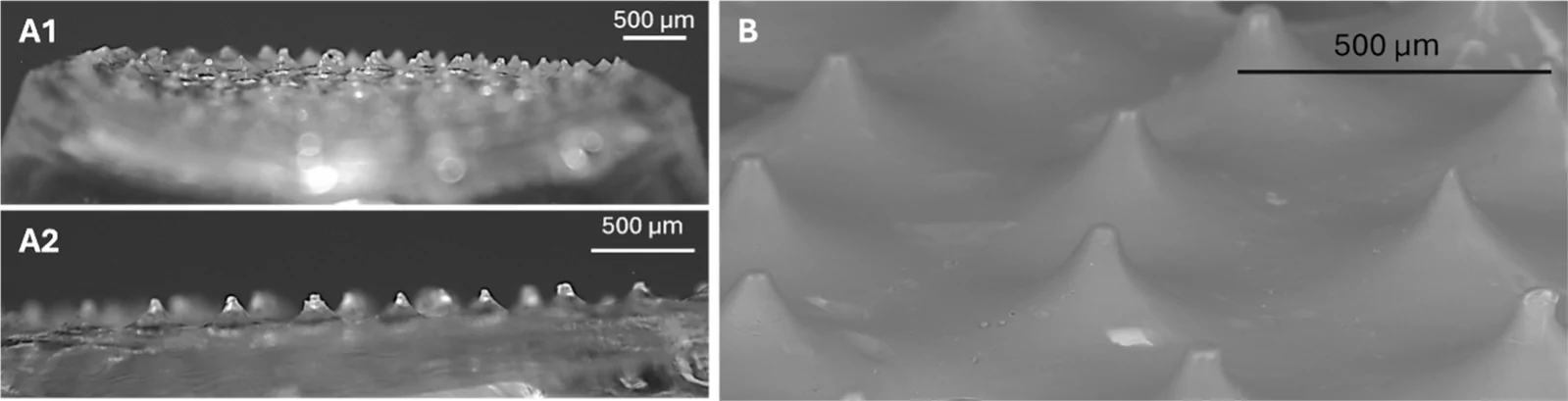
Typical chips of MNs fabricated by the molding procedure in PLGA material observed by (A1, A2) optical microscope and by (B) SEM. A1 shows a large view over the whole surface of the chip, while A2 shows a closer view over a single row of microneedles, where the shape appears well replicated.

In this work, we functionalized this kind of MN chip for the first time with the in-house synthesized P52 peptide for the specific TNF-α detection in simulated interstitial fluid. Figure 2 A-C shows the schematic view of the protocol used for this purpose, consisting of a 3D sandwich-like assay as explained in the following. The washing steps described in the methods section are not illustrated for clarity of presentation. For the same reason, peptide binding is represented only at MN tips, but it is expected to be distributed along the MN surface.

**Figure 2:**
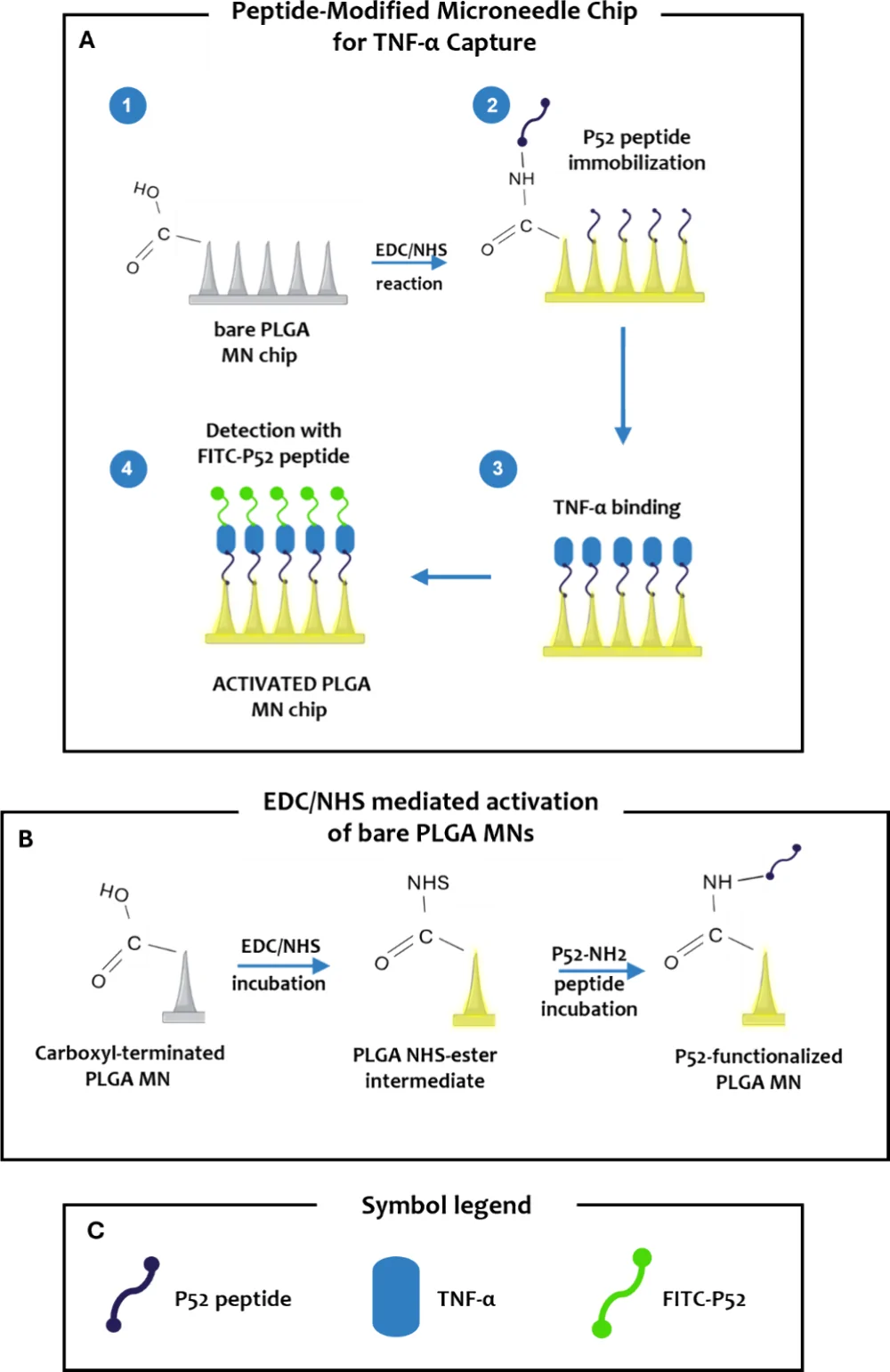
Schematic representation of the P52-functionalized PLGA microneedle platform for TNF-α detection. (A) Main steps of the sandwich-like fluorescence assay: (1) bare PLGA MNs; (2) EDC/NHS-mediated surface activation followed by P52 immobilization; (3) TNF-α binding to surface-immobilized P52; and (4) fluorescence detection through incubation with FITC-P52. (B) Enlarged schematic of the proposed EDC/NHS-mediated coupling pathway. Accessible carboxyl groups on PLGA are converted into NHS-ester intermediates, which can react with primary amino groups of P52, promoting stable peptide immobilization through the expected amide-linkage formation. (C) Symbol legend for P52 peptide, TNF-α, and FITC-P52.

First, the MN chip is activated by a standard EDC/NHS reaction (0.1 and 0.2 M, respectively), where EDC activates the carboxyl groups −COOH on PLGA, forming an unstable intermediate. Then, NHS stabilizes this intermediate by converting it into an NHS-ester which is more reactive and has a longer lifetime. For brevity, we call ‘activated’ the MNs after this EDC/NHS treatment. Successively, the P52 is added at 1 mg/mL, so that its primary amines readily attack the NHS-ester promoting the formation of amide linkages between P52 and the PLGA backbone. EDC/NHS coupling was selected as a well-established zero-length bioconjugation strategy for the immobilization of amine-containing peptides onto carboxyl-bearing polymeric surfaces. A similar approach has previously been successfully employed for peptide grafting onto poly­(acrylic acid)-functionalized PDMS, where the formation of amide linkages was supported by infrared spectroscopy and HPLC analysis.^62^ More recently, the same coupling approach was employed to conjugate the GE11 peptide to PLGA nanoparticles through the reaction between PLGA carboxyl groups and peptide amino groups, with FTIR analysis supporting the proposed amide-bond formation.^63^ Although these studies employed different polymeric materials and geometries, they support the chemical plausibility of EDC/NHS-mediated peptide immobilization on accessible carboxyl groups of PLGA.

After activation and functionalization with P52, replicated MN chips are then incubated with samples of the target molecule TNF-a at different dilutions in PBS and artificial ISF (0, 0.11, 0.23, 0.46, 0.69, 1.84, and 5.74 pM). The detection of the target molecule is then achieved by a final incubation with a FITC-labelled form of the P52 peptide (FITC-P52, 1 mg/mL), which binds specifically to the TNF-a molecules immobilized on the MN chip. A calibration curve is then retrieved through the evaluation of the resulting fluorescence signal intensity for each TNF-a sample.

### Peptide Immobilization on Microneedles

In this section, before starting the assay protocol for the TNF-α detection, we evaluated the stability of P52 immobilization on the MN chips by using a FITC-labelled form of the peptide P52. In particular, we followed the process steps illustrated in Figure 3 using two kinds of MN chips: (1) non-activated MN chip, namely the bare one not subjected to the EDC/NHS process submerged in the FITC-P52 solution; (2) activated MN chip, namely the one subjected to the EDC/NHS treatment and then incubated with the FITC-P52 solution. This comparison allowed us to distinguish the contribution of non-specific peptide adsorption from the enhanced immobilization promoted by surface activation.

**Figure 3:**
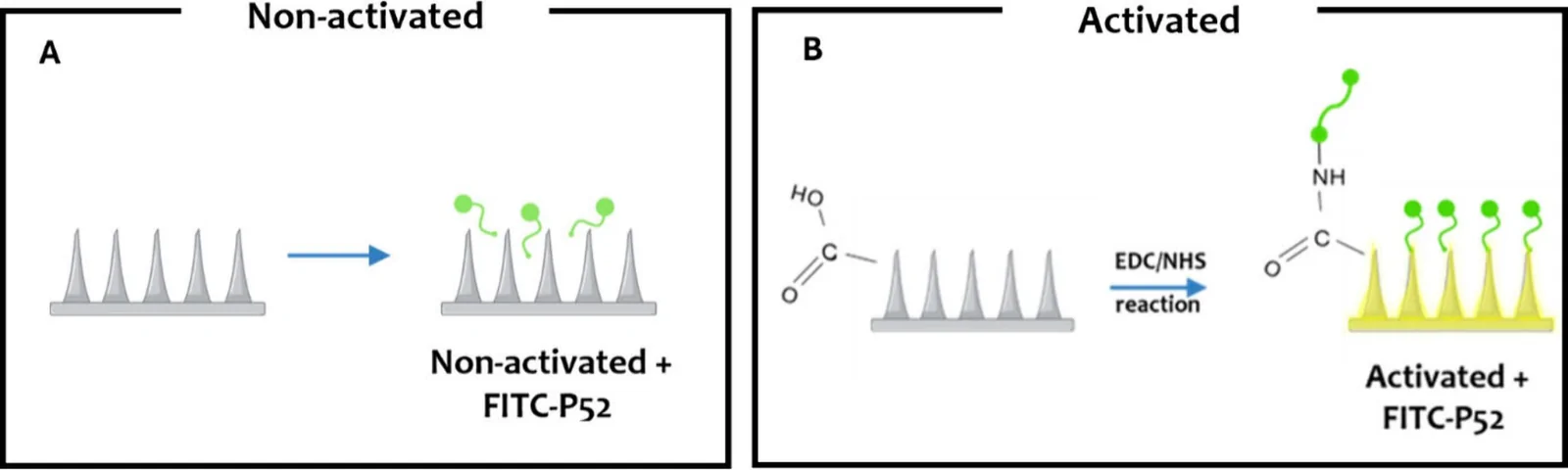
Schematic representation of the steps performed to obtain the non-activated MN chip with nonspecific adsorption of the FITC-P52 peptide molecules (left), and of the steps to obtain the activated MN chip with enhanced immobilization of the fluorescent peptide after EDC/NHS chemical activation (right).

We incubated both chips with the FITC-P52 peptide at a concentration of 1 mg/mL at 37° C and 100 rpm overnight. As mentioned before, the incubation of the non-activated chip with P52-FITC was used for the evaluation of the non-specific interactions between the peptide and the PLGA surface (Figure 3-A). Conversely, in the activated chip, EDC/NHS treatment promoted the formation of reactive NHS-esters from the carboxylic groups exposed on the PLGA surface. These intermediates can react with the free amino groups of P52 (Figure 3-B), leading to the formation of more stable interactions as visible by the fluorescence analysis detailed in the following section. After washing and drying, we recorded the fluorescence microscope images of three kinds of chip samples where the incubation is intended with the FITC-P52: (1) untreated chip, namely the non-activated MN chip without any peptide incubation, corresponding to what we call “bare”; (2) non-activated chip after incubation, corresponding to what we call “non activated + FITC-P52”; (3) activated chip after incubation, corresponding to what we call “activated + FITC-P52”. Figure 4A1-A3 shows the typical fluorescence images for these three kinds of chips, as indicated by the labels. We compared the fluorescence intensities recorded for the three kinds of chips. Figure 4-B shows the mean fluorescence intensities evaluated from the fluorescence microscope images.

**Figure 4:**
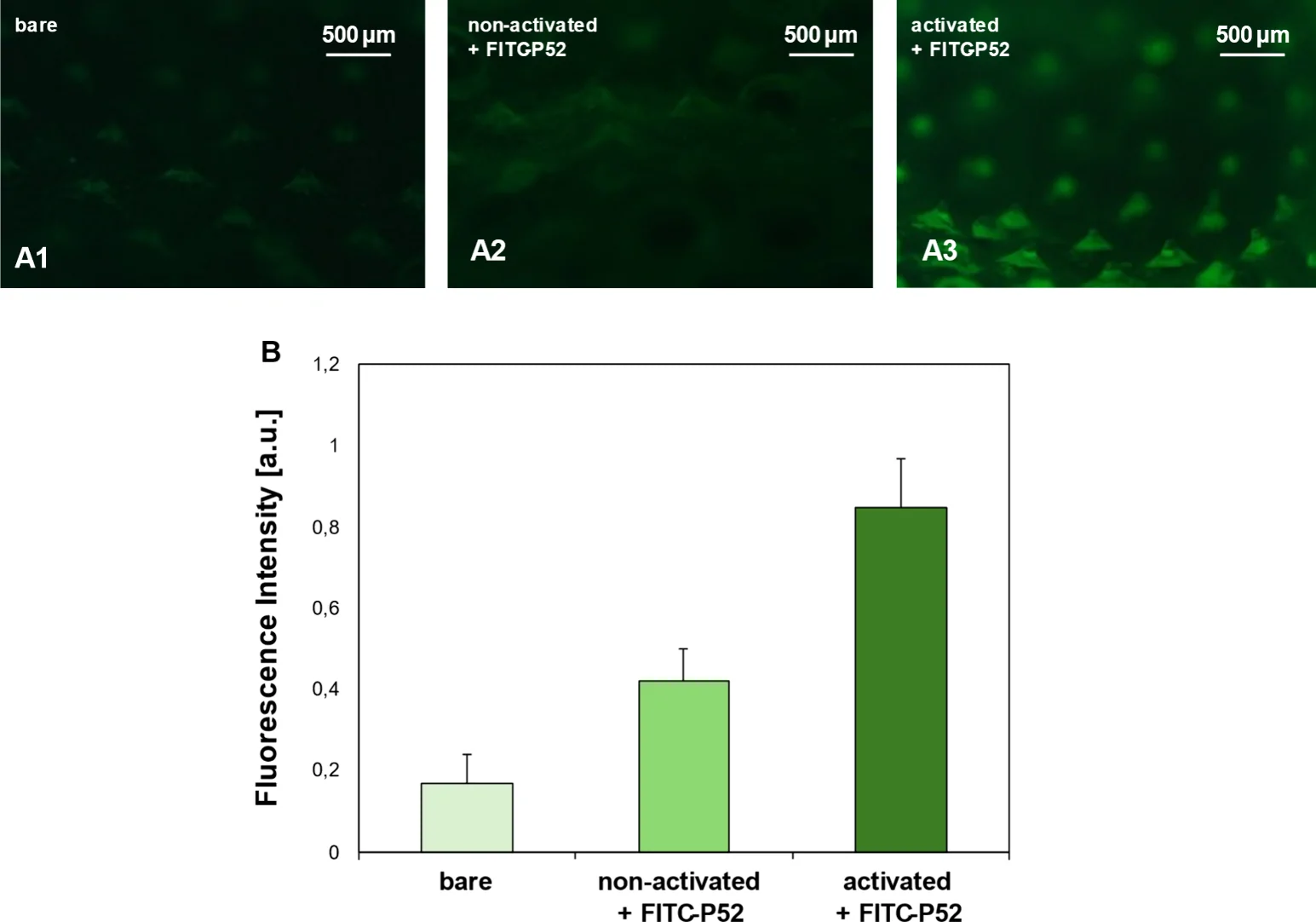
(A1)-(A3) Typical fluorescence images of three kinds of MN chips used for evaluating the stability of the FITC-P52 peptide binding on the MN surface. A1 shows the fluorescence image of the background signal, namely that of the bare MN chip. A2 corresponds to the control signal, namely the fluorescence image of the non-activated MN chip after incubation with P52-FITC. A3 corresponds to the peptide signal, namely the fluorescence image from the MN chip activated and incubated properly with the P52-FITC peptide. (B) ImageJ measurements of the mean fluorescence intensity (normalized value) of fluorescence microscope images after P52-FITC incubation, where the mean values and the standard deviations were evaluated over three replicas of the experiment.

The mean intensities and standard deviations were evaluated over three replicas of the experiment, and for each experiment, a total of 20 needles were analyzed. Quantitative analysis performed using ImageJ after washing steps provided indirect evidence of stable P52 immobilization on the activated PLGA surface. The weak signal observed in the bare PLGA microneedles is attributed to the background optical response of the polymeric substrate under the selected fluorescence imaging conditions, which may arise from a combination of intrinsic polymer background, light scattering, and surface reflection. Similar background optical interference from PLGA-based biomaterials during fluorescence imaging has been previously reported.^64^ The mean fluorescence intensity corresponding to the “non-activated + FITC-P52” appears on average 60% higher than the bare chip, indicating that a measurable fraction of the peptide was retained through non-specific adsorption. This behavior may result from hydrophobic, electrostatic, or hydrogen-bonding interactions between FITC-P52 and the PLGA surface.^65,66^ However, the “peptide signal” generates on average a mean intensity 50% higher than that of the non-activated and around 80% higher than that of bare MNs. The enhanced signal retained after repeated washing therefore supports the increased amount and stability of surface-bound peptide following EDC/NHS activation. Figure S1 shows the UV-VIS spectra of the FITC-P52 peptide solution used for the incubation of the activated MNs. The characteristic adsorption peaks (at 280 nm for peptide and 490 nm for FITC, respectively) decreased in the post-incubation supernatant compared with the initial peptide solution. This reduction indicates that part of the FITC-P52 peptide was removed from the solution and provides indirect evidence of EDC/NHS-mediated immobilization. Taken together, the significantly higher fluorescence retained by the activated MNs after washing, together with the depletion of FITC-P52 from the incubation solution, demonstrates that EDC/NHS activation provides a more efficient and stable functionalization strategy than passive adsorption alone. Establishing reproducible P52 immobilization was essential for the subsequent TNF-α sandwich assay, since variations in the density or stability of the capture element could directly affect target binding, fluorescence intensity, and sensor reproducibility.

#### TNF-α Binding in PBS and Artificial Subcutaneous ISF

Once the ability of the MNs to immobilize P52 was validated, the detection capabilities of our MNs-based sensor in PBS buffer at different TNF-α concentrations were assessed. The sensor was first functionalized with non-fluorescent P52 (1 mg/mL), incubated with varying concentrations of TNF-α, and finally exposed to FITC-labeled P52 peptide (1 mg/mL). The curve obtained in PBS 1X exhibited a rapid initial increase in fluorescence, reaching a plateau at very low concentration (∼1–2 pM), indicative of a high affinity of the peptide for TNF-α. The initial signal increase can be attributed to the progressive occupation of the available P52 recognition sites on the MN surface, whereas the subsequent plateau is consistent with the saturation of a finite number of surface-accessible binding sites. This behavior is particularly relevant for cytokine detection because these biomarkers are generally present at low concentrations in biological fluids and therefore require highly efficient capture systems. The one site – specific binding model well explained this behavior, with a correlation coefficient of R^2^ = 0.87 in PBS (Figure 5-A). The same concentration points were detected in a more complex and realistic environment, namely the simulated subcutaneous ISF. In this case, the increase in fluorescence intensity was less pronounced, i.e. smaller signal change per unit concentration; in fact, the same variations in concentrations corresponded to a lower increase in the signal (Figure 5-B). This demonstrated less sensitivity of the assay, which could be attributed to the presence of interfering components in the simulated biofluid, like lipids, salts, and albumin protein that can non-specifically adsorb to the sensor surface.^1^ The saturation value was still achieved, but at higher concentrations (∼5 pM), and the ‘One site – Specific binding’ model fit well the data, with a better correlation coefficient of R^2^ = 0.95. The reduced slope and the shift of the saturation region toward higher TNF-α concentrations indicate that the simulated ISF matrix partially affected the analytical response of the platform. Matrix-dependent effects may result from differences in ionic strength, pH, analyte diffusion, or non-specific interactions between fluid components and the peptide-functionalized PLGA surface. Therefore, at this stage the observed behavior is more appropriately described as a general matrix effect rather than being attributed to an individual interfering species.^67^

**Figure 5:**
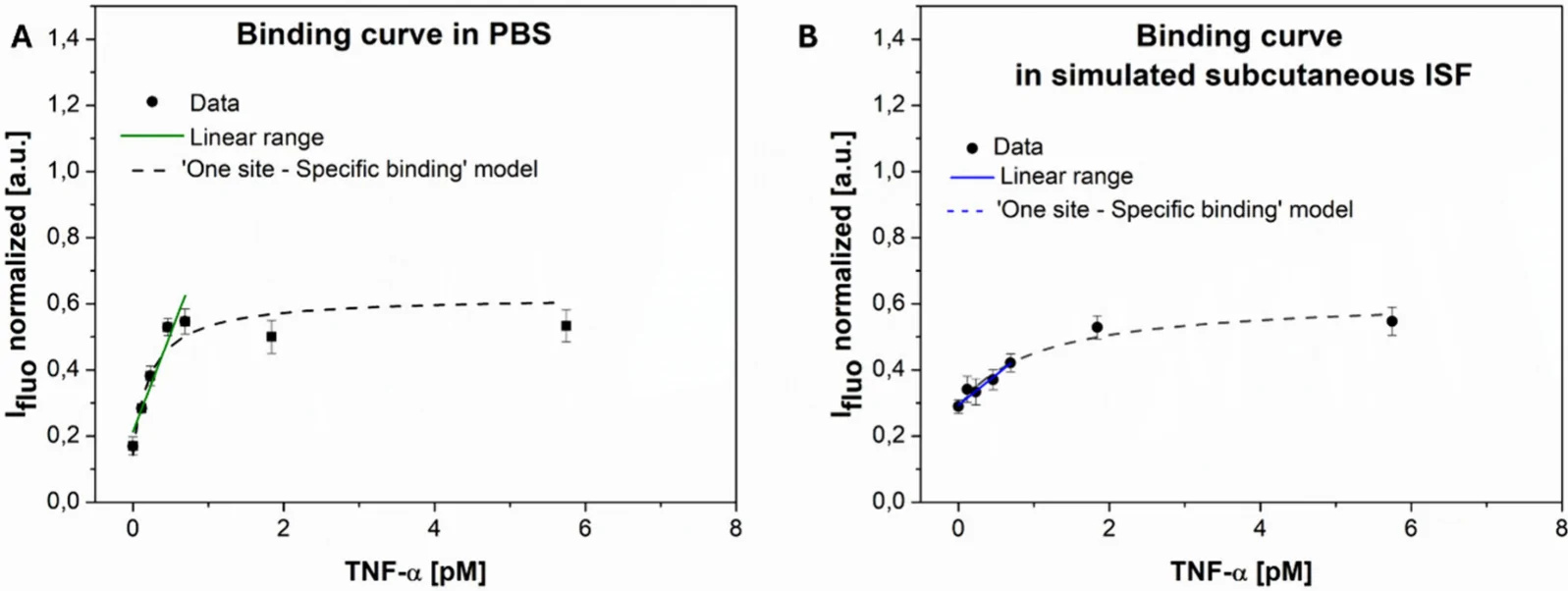
Binding curves of P52 and TNF-α obtained in (A) PBS 1X buffer and (B) simulated subcutaneous ISF. Experimental data are shown as circles (mean ± standard deviation). The solid line highlights the linear fit considered for LoD and LoQ calculations. The dashed line corresponds to the nonlinear fit using the ‘One site - Specific binding’ model.

Table S1 summarizes the parameters estimated from the model fitting. The peptide showed high affinity for TNF-α in both media, with LOD and LOQ in the picomolar range. LOD and LOQ were equal to 0.17 pM and 0.51 pM in PBS, and to 0.13 pM and 0.39 pM in ISF while K_D_ was 0.2 pM in PBS and 1.0 pM in ISF, as reported in Table 2 for a comparison. Detection limits were marginally lower in simulated ISF; however, the reduced slope in the linear range (Figure 5-B, blue line) implies lower sensitivity, probably due to the ISF matrix, as mentioned previously. Accordingly, the principal finding is that the sensor achieved good performance also in simulated ISF rather than in PBS, preserving low-picomolar TNF-α detection capability even if in the presence of matrix-dependent interference. The slightly lower K_D_ measured in PBS indicates higher apparent affinity in PBS than in ISF, consistent with components interfering with binding. Although the performance is optimal in PBS, it remains highly effective in simulated ISF, which better mimics real biological conditions. These results support the robustness and reliability of the sensor even in complex fluids. From the binding modeling, K_D_ values were also obtained. As a negative control, MNs were functionalized with the P11 peptide (1 mg/mL) as the capture element, which is not specific for TNF-α. Three levels of analyte (0 pM, 0.11 pM, 1.84 pM, 5.74 pM) were detected in ISF following the procedure already described. The UV-vis spectrum of the FITC-P11 solution employed for the incubation of the detection element is reported in Figure S2. Under these conditions, the fluorescence signal exhibited a weak correlation with the analyte concentration, and the fluorescence data did not follow a binding trend compatible with the ‘One site – Specific binding’ model (Figure S3). As a result, the reliable value of the K_D_ could not be determined. The detected fluorescence may be attributed to non-specific interactions between the ligand and the target analyte (electrostatic, adsorption) or to background signal. The absence of a reproducible and saturable concentration-dependent response supports the sequence-dependent recognition of TNF-α by P52 and confirms the importance of the peptide sequence in generating the analytical signal. Nevertheless, P11 represents a negative peptide control rather than a complete evaluation of analytical selectivity. Future developments of the proposed methodology will involve potentially interfering cytokines or abundant biological proteins in order to fully assess cross-reactivity in physiologically relevant samples. To provide a complementary and more traditional quantitative evaluation of P52 binding to TNF-α, BLI assays were conducted. The P52 peptide was synthesized in the biotinylated–pegylated form and immobilized on a streptavidin-coated biosensor. Kinetic experiments, carried out at diverse TNF-α concentrations in the range of 0.5–4 nM (Figure S4), provided dose–response signals, allowing the estimation of a K_D_ value equal to 3.6±0.3 nM. Compared to BLI, the MN-based setup achieves stronger binding signals as reflected by the lower K_D_ (Table 2). This effect likely arises from the sandwich assay format and the confined geometry of the microneedles, which increased local peptide concentration and facilitated multivalent interactions, ultimately leading to higher sensitivity compared to the direct binding measured by BLI.

**Table 2:** Comparison of the P52 Assays Performed in PBS 1X and Simulated Subcutaneous ISF

**FLUID**	**LOD**	**LOQ**	**R** ^**2**^ **for One site – Specific binding model**	**K**_**D**_
PBS 1X buffer	**(0.17 ± 0.03) pM**	**(0.51 ± 0.08) pM**	**0.87**	**(0.2 ± 0.1) pM**
Artificial subcutaneous ISF	**(0.13 ± 0.01) pM**	**(0.39 ± 0.04) pM**	**0.95**	**(1.0 ± 0.3) pM**

#### Microneedles Mechanical Strength; Penetration in Collagen Matrix

Compression tests were carried out on bare PLGA and PLGA + P52 MNs arrays to evaluate their mechanical performance under axial loading. For each group, three independent tests were performed, and in all cases, the MNs remained structurally intact, with no evidence of buckling, fracture, or permanent deformation. The force–displacement curves obtained during testing exhibited a nearly linear profile, with no sudden variations in slope (Figure 6-A, B), suggesting that the MNs behaved elastically within the range of applied forces. Moreover, no differences were observed between bare PLGA and PLGA + P52, demonstrating that the immobilization of the peptide did not compromise the properties of the MNs. This linear trend is indicative of a stable mechanical response and is consistent with the elastic regime commonly reported for PLGA-based MNs under similar loading conditions.^68^ The absence of mechanical failure demonstrates the robustness of the MN design and confirms that the fabrication process yielded geometrically uniform and mechanically reliable structures. Mechanical integrity is a crucial parameter, as MNs must penetrate the stratum corneum without bending or breaking during insertion.^69^ Confocal laser scanning microscopy demonstrated successful insertion of 300 μm MNs into the 400 μm-thick 3D collagen matrix (Figure S5 A-C, Movie S1). Z-stack reconstructions and 3D rendering allowed clear visualization of the MN geometry and the local deformation of collagen fibers surrounding the insertion site (Figure S5 A-C). The collagen fibrillar network and its rearrangement upon MN insertion were revealed by SHG imaging. Z-stack analysis estimated a penetration depth of approximately 250 μm (Movie S1, 3D reconstruction of the collagen Z-stack 30 min after needle insertion). These results demonstrate the effective penetration of 380 μm MNs into a collagen-based, physiologically relevant 3D environment. Together with their preserved mechanical integrity, this penetration capability supports the potential use of the functionalized MNs in minimally invasive sensing applications. In our previous study the cytocompatibility of PLGA MNs was demonstrated also for PLGA MNs subjected to EDC/NHS activation and subsequent functionalization with antibodies and gold nanoparticles. The results showed preserved cell viability after 24 h of incubation in SK-N-BE(2) neuroblastoma cells, providing experimental evidence that the PLGA MN platform can retain cytocompatibility after carbodiimide-mediated surface activation, functionalization, and the associated washing procedures.^19^ Although the previous functionalized platform employed antibodies and gold nanoparticles rather than P52, the use of the same polymeric substrate and the same type of EDC/NHS activation provide relevant supporting evidence regarding the compatibility of the surface-treatment procedure. Nevertheless, dedicated cytocompatibility testing of the present P52-functionalized MNs will be required to confirm the biological safety of the final sensing configuration.

**Figure 6:**
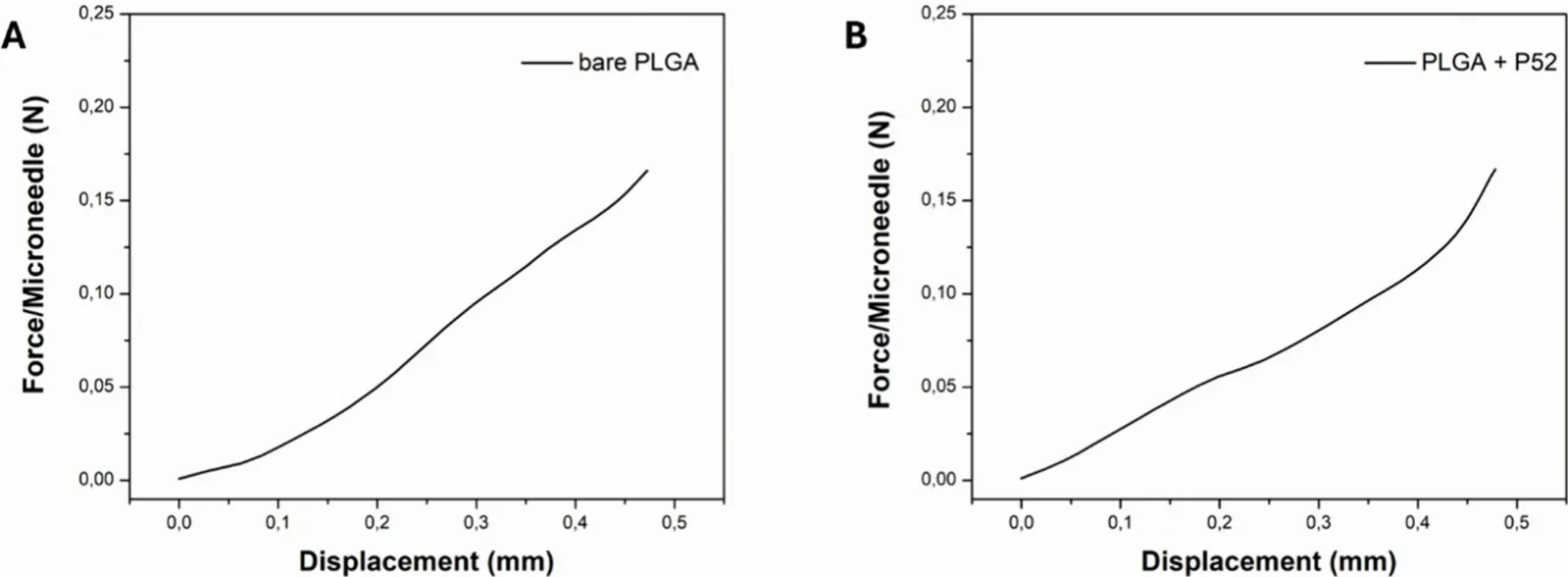
Force vs displacement curve under compression for (A) bare PLGA and (B) PLGA + P52 MN arrays.

The penetration results should also be interpreted considering the limitations of the experimental model. Collagen gels reproduce selected structural features of the dermal extracellular matrix but do not reproduce the multilayer organization, toughness, hydration state, and barrier function of intact skin. Therefore, this experiment should be presented as a proof of penetration into a dermal-like fibrillar environment rather than definitive evidence of transdermal insertion. Future studies using ex vivo skin and controlled application forces will be necessary to determine insertion efficiency under more physiologically representative conditions.

## Conclusion

This work demonstrates that the in-house synthesized P52 peptide can be effectively immobilized on PLGA MNs via EDC/NHS chemistry and used as a robust biorecognition element for TNF-α detection. The main novelty lies in combining the stability, reproducibility, and ease of functionalization of a short synthetic peptide with the three-dimensional surface and potential transdermal accessibility of a polymeric MN array. The platform retained low-picomolar detection capability in simulated ISF, while peptide functionalization did not compromise the mechanical response or penetration capability of the MNs. Synthetic peptides with their small size and well-defined chemical composition proved to facilitate reproducible synthesis and high-density immobilization on polymeric surfaces. We showed that translating peptide-based molecular recognition to MN biosensors included several technical challenges like stable peptide immobilization while preserving the accessibility of the recognition sequence, maintaining analytical sensitivity in complex biological matrices, and ensuring that the surface-functionalization procedure does not compromise MN mechanical integrity or penetration capability. From a practical perspective, replacing conventional antibody recognition elements with a synthetic peptide may reduce production costs, improve batch-to-batch reproducibility, and facilitate the scalable fabrication and storage of MN-based biosensors. The proposed approach will therefore provide a promising basis for the development of compact and minimally invasive systems for monitoring inflammatory biomarkers in ISF. We are confident that our results would inspire additional studies on the use of synthetic peptide and in the analysis of selectivity against potentially interfering cytokines and abundant biological proteins. Additional studies would need also to evaluate sensor performance in ex vivo skin and real ISF samples and investigate stability and assay reproducibility over extended periods. Integration with a portable fluorescence readout and expansion toward multiplex cytokine detection will also be important steps toward future commercial and mass production point-of-care applications.

## Supplementary Material





## Notes

Z-stack acquisition of the MN penetration in 3D collagen matrix (AVI)
UV-vis spectra of fluorescent peptides, parameters estimated from the ‘One site – Specific binding’ model fitting, negative control experiment, results of Bio Layer Interferometry (BLI), confocal imaging of microneedle penetration into the collagen matrix (PDF)
