# Supplementary material for: Combination of Polymeric Microneedles with Specific Peptide for TNF‑α Sensing Via Noninvasive Detection in Interstitial Fluid
Source: ACS Omega. 2026 Sep 16;11(38):57962–73. doi: 10.1021/acsomega.6c07406 (PMC13629057; doi:10.1021/acsomega.6c07406)
Supplement: Supplementary file 1 [file ao6c07406_si_002.pdf]

Combination of Polymeric Microneedles with specific peptide for TNF-α sensing via Noninvasive Detection in Interstitial Fluid

Anna Palma[a,b], Concetta Di Natale[a,], Daniele Tammaro[a], Elena Lagreca[a], Alessia Cugudda[c], Sara La Manna[c], Vincenzo Ferraro[d], Veronica Vespini[b], Simonetta Grilli[b], Pier Luca Maffettone[a], Sara Coppola[b,*]*

[a] Department of Chemical, Materials, and Industrial Production Engineering (DICMaPI), University of Naples Federico II, 80125 Naples, Italy

[b] Institute of Applied Sciences and Intelligent System (CNR-ISASI), Via Campi Flegrei 34, 80078 Pozzuoli (NA), Italy

[c] Department of Pharmacy, University of Naples Federico II, 80131 Naples, Italy

[d] Department of Engineering and Architecture, University of Parma, 43124, Parma, Italy

[*] Corresponding authors: concetta.dinatale@unina.it ; sara.coppola@cnr.it

Supplementary material

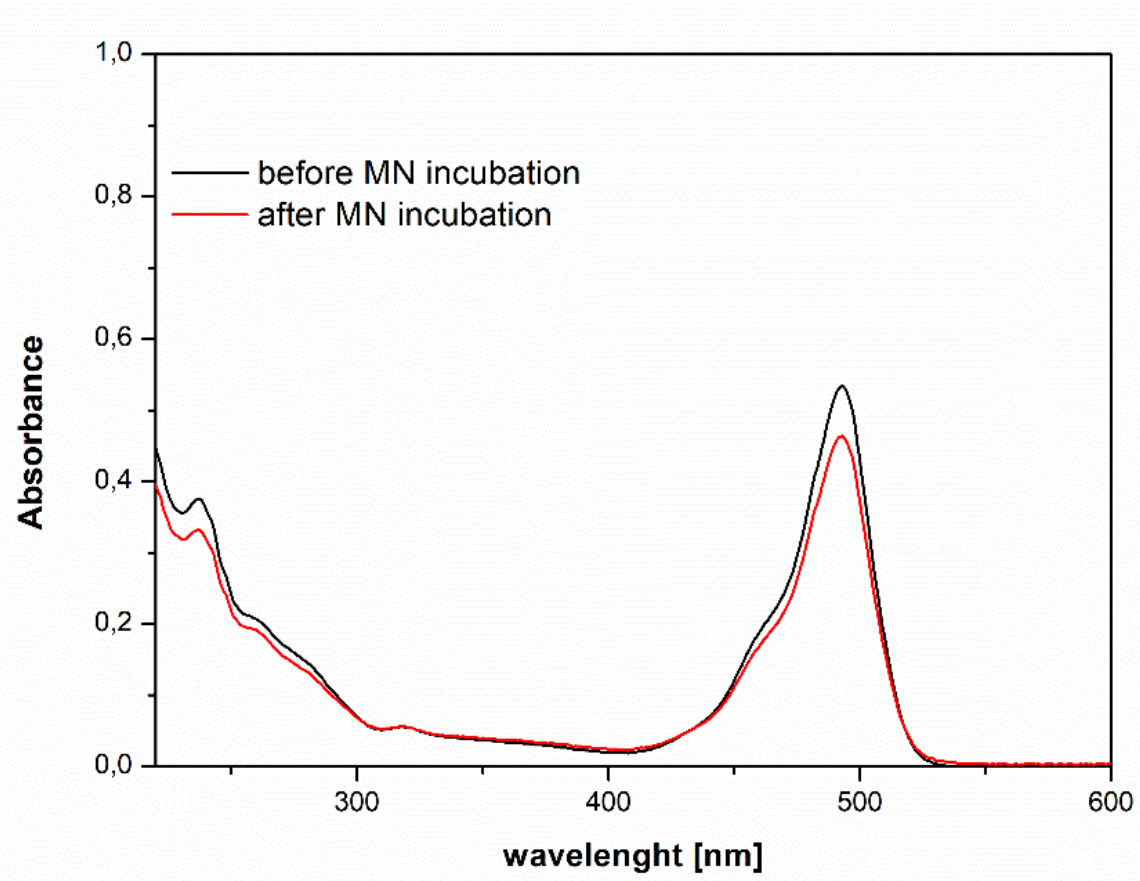

Figure S1. *UV-VIS spectra of the FITC-P52 peptide solution before and after incubation with the activated MN chip, corresponding to the black and red lines, respectively. The characteristic UV-vis peaks (at 280 nm for peptide and 490 nm for FITC) of the supernatant clearly decreases after MN incubation compared to the one of the mother solution before any incubation step. Both spectra refer to a 1:10 dilution.*

Table S1. Parameters estimated from the 'One site – Specific binding' model fitting of the experimental data obtained in PBS 1X buffer and simulated ISF. The equation was: $Y = Y_{NS} + \frac{B_{max} \cdot x}{K_D + x}$.

FLUID	R^2 for One site – Specific binding model	B_{MAX}	K_D	Y_{NS}
PBS 1X buffer	**0.87**	**(0.48 ± 0.08) pM**	**(0.2 ± 0.1) pM**	**(0.14 ± 0.05) pM**
Artificial subcutaneous ISF	**0.95**	**(0.33 ± 0.04) pM**	**(1.0 ± 0.3) pM**	**(0.29 ± 0.01) pM**

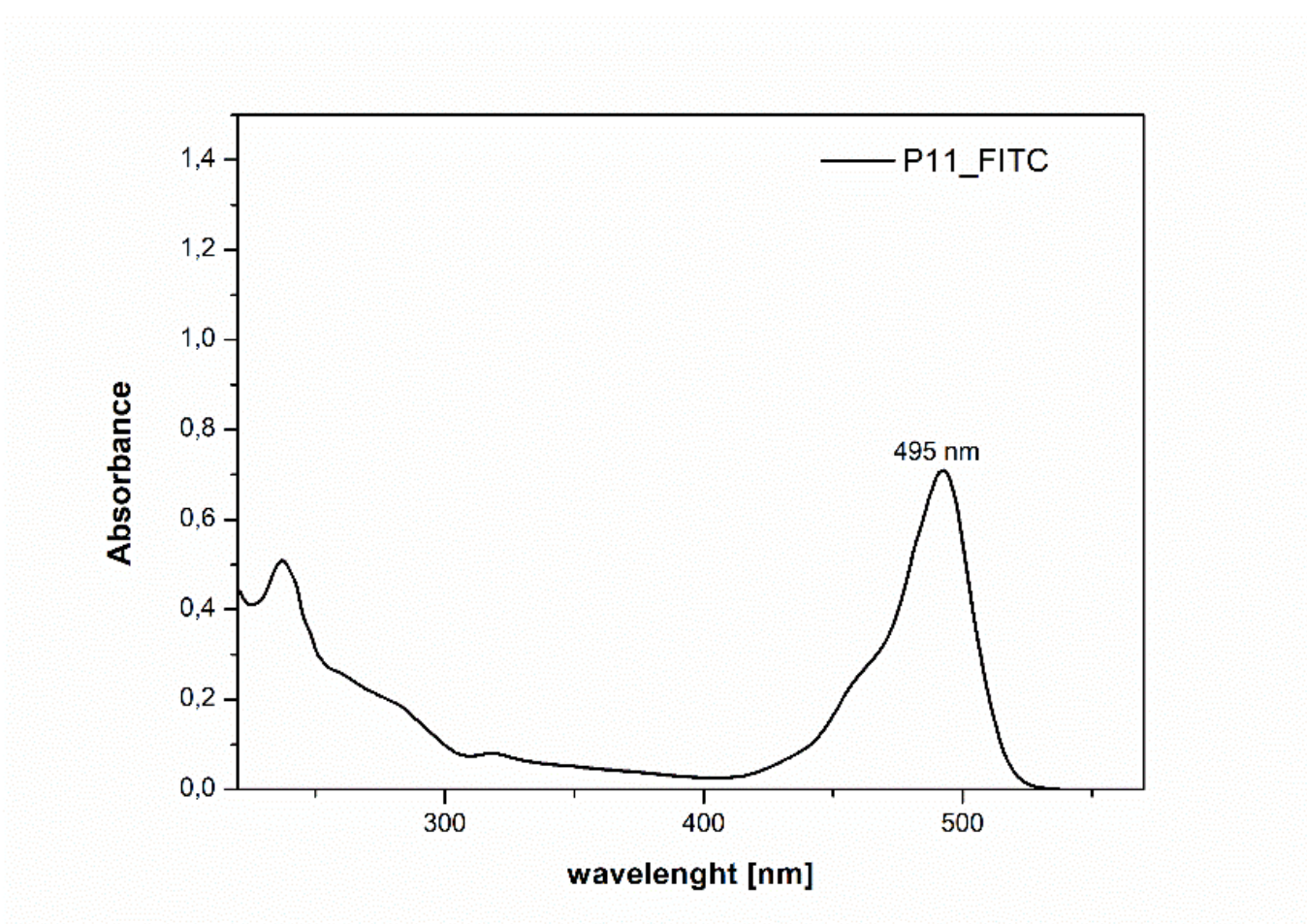

Figure S2. *UV-vis spectrum of the detection peptide FITC-P11 used as the negative control of the calibration curve in simulated ISF.*

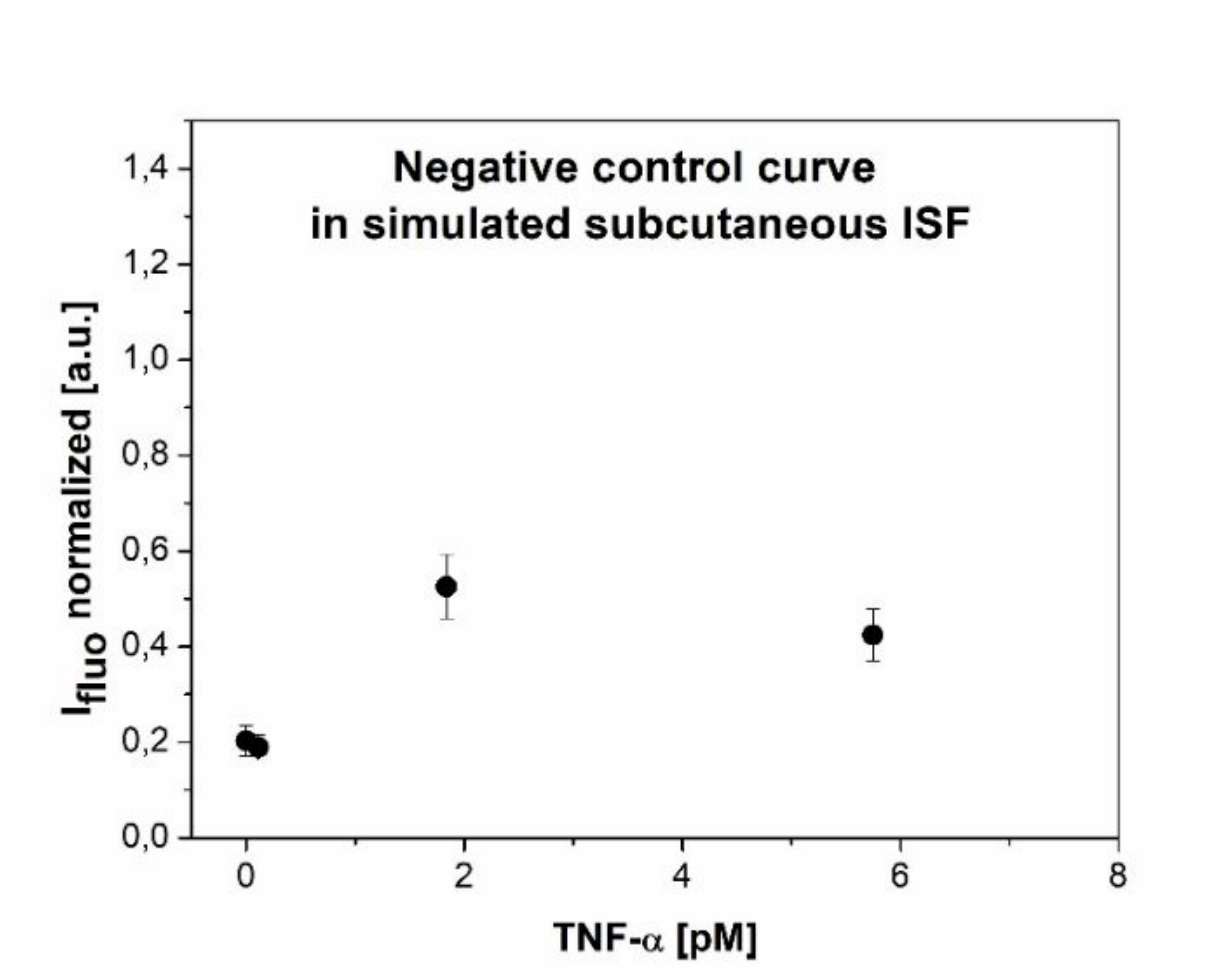

Figure S3. *Negative control of the signal detection obtained using FITC-P11 peptide in simulated subcutaneous ISF.*

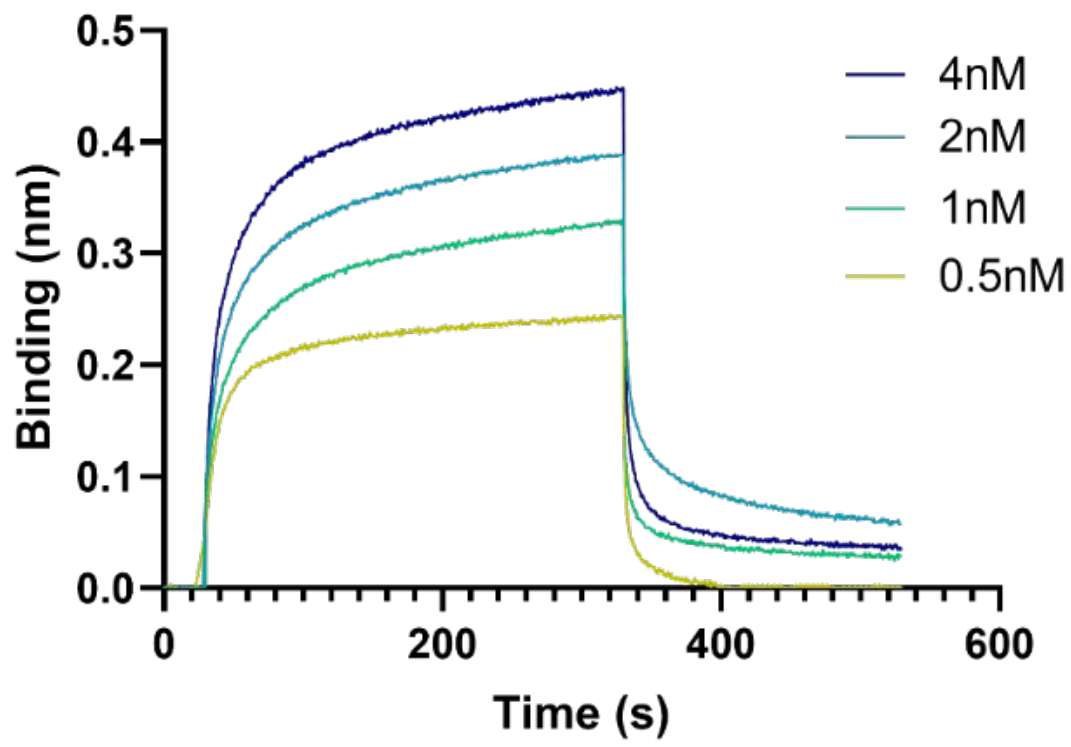

Figure S4. *BLI traces for binding of immobilized P52 peptide to TNFα.*

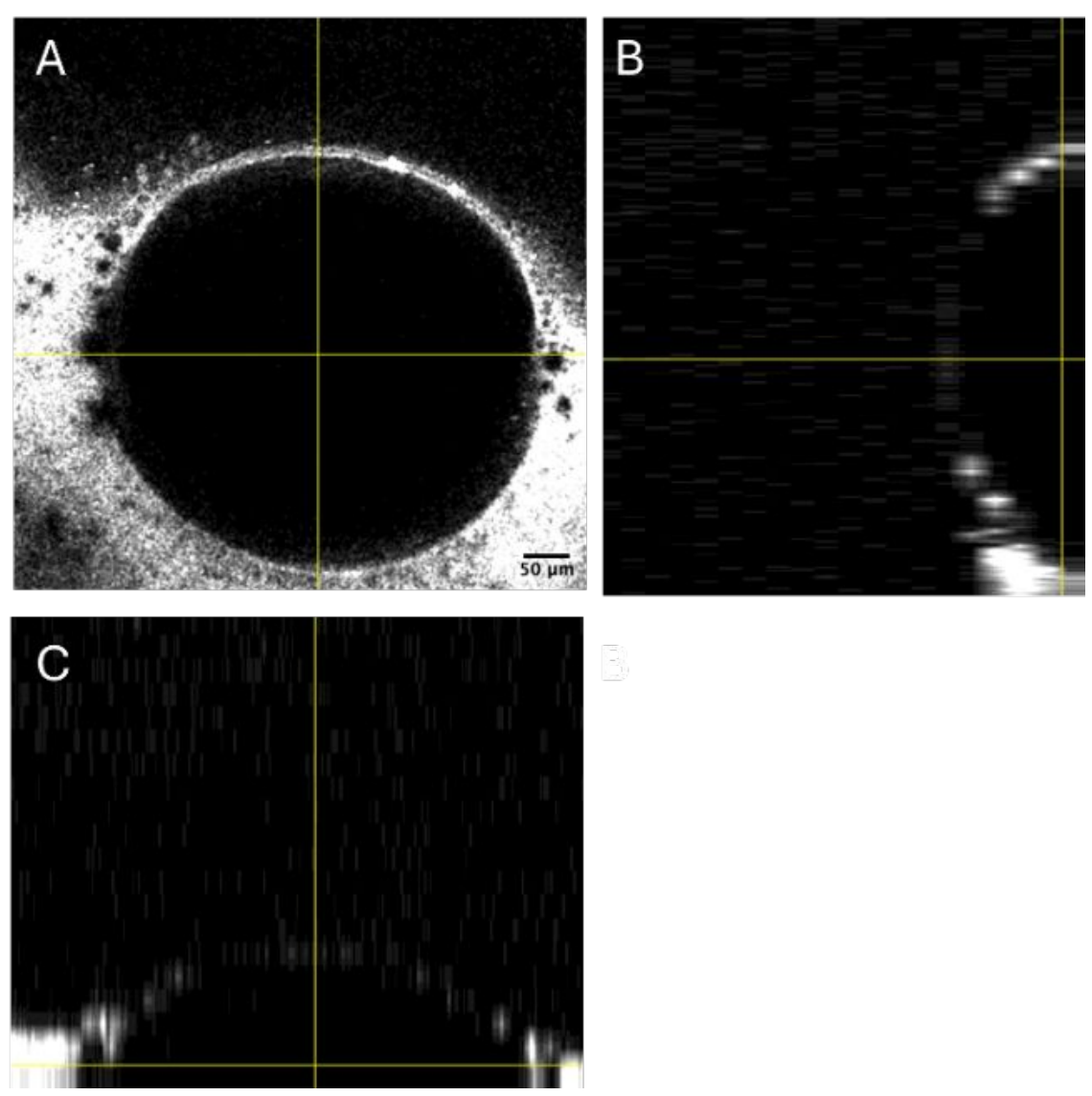

Figure S5. *Orthogonal views of MNs implantation in a 3D collagen matrix 50 µm scale bar: (A) SGH signal for PLGA autofluorescence and collagen fiber, (B) trasversal section along y axis, and (C) transversal section along x axis.*

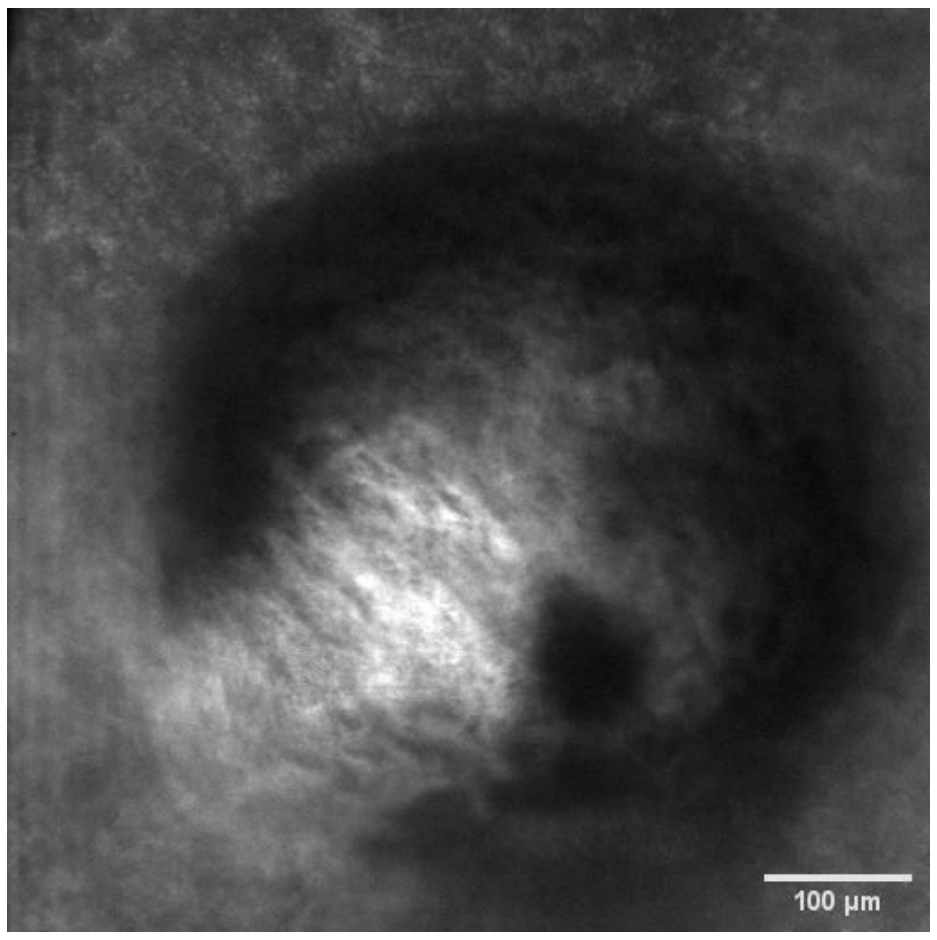

***Movie 1**. Z-stack acquisition of the MN penetration in 3D collagen matrix.*
